# Fermented Rice Bran Supplementation Prevents the Development of Intestinal Fibrosis Due to DSS-Induced Inflammation in Mice

**DOI:** 10.3390/nu13061869

**Published:** 2021-05-30

**Authors:** Afifah Zahra Agista, Tubagus Bahtiar Rusbana, Jahidul Islam, Yusuke Ohsaki, Halima Sultana, Ryota Hirakawa, Kouichi Watanabe, Tomonori Nochi, Ardiansyah, Slamet Budijanto, Suh-Ching Yang, Takuya Koseki, Hisashi Aso, Michio Komai, Hitoshi Shirakawa

**Affiliations:** 1 Laboratory of Nutrition, Graduate School of Agricultural Science, Tohoku University, 468-1 Aramaki Aza Aoba, Aoba-ku, Sendai 980-8572, Japan; agista@g-mail.tohoku-university.jp (A.Z.A.); tbbahtiar@untirta.ac.id (T.B.R.); islam.jahidul.a5@tohoku.ac.jp (J.I.); yusuke.ohsaki.a4@tohoku.ac.jp (Y.O.); sultana.halima.d4@tohoku.ac.jp (H.S.); mkomai@m.tohoku.ac.jp (M.K.); 2 Department of Food Technology, Faculty of Agriculture, University of Sultan Ageng Tirtayasa, Serang 42110, Indonesia; 3 International Education and Research Center for Food Agricultural Immunology, Graduate School of Agricultural Science, Tohoku University, 468-1 Aramaki Aza Aoba, Aoba-ku, Sendai 980-8572, Japan; watakoh@g-mail.tohoku-university.jp (K.W.); tomonori.nochi.a5@tohoku.ac.jp (T.N.); asosan@tohoku.ac.jp (H.A.); 4 Laboratory of Functional Morphology, Graduate School of Agricultural Science, Tohoku University, 468-1 Aramaki Aza Aoba, Aoba-ku, Sendai 980-8572, Japan; r-hirakawa@g-mail.tohoku-university.jp; 5 Department of Food Technology, Universitas Bakrie, Jakarta 12920, Indonesia; ardiansyah.michwan@bakrie.ac.id; 6 Faculty of Agricultural Engineering and Technology, IPB University, Bogor 16680, Indonesia; slamet.budijanto@gmail.com; 7 School of Nutrition and Health Sciences, Taipei Medical University, Taipei 110, Taiwan; sokei@tmu.edu.tw; 8 Faculty of Agriculture, Yamagata University, Tsuruoka 997-8555, Japan; tkoseki@tds1.tr.yamagata-u.ac.jp

**Keywords:** colitis, dextran sodium sulfate, fermented rice bran, intestinal fibrosis, intestinal inflammation

## Abstract

Fermented rice bran (FRB) is known to protect mice intestines against dextran sodium sulfate (DSS)-induced inflammation; however, the restoration of post-colitis intestinal homeostasis using FRB supplementation is currently undocumented. In this study, we observed the effects of dietary FRB supplementation on intestinal restoration and the development of fibrosis after DSS-induced colitis. DSS (1.5%) was introduced in the drinking water of mice for 5 days. Eight mice were sacrificed immediately after the DSS treatment ended. The remaining mice were divided into three groups, comprising the following diets: control, 10% rice bran (RB), and 10% FRB-supplemented. Diet treatment was continued for 2 weeks, after which half the population of mice from each group was sacrificed. The experiment was continued for another 3 weeks before the remaining mice were sacrificed. FRB supplementation could reduce the general observation of colitis and production of intestinal pro-inflammatory cytokines. FRB also increased intestinal mRNA levels of anti-inflammatory cytokine, tight junction, and anti-microbial proteins. Furthermore, FRB supplementation suppressed markers of intestinal fibrosis. This effect might have been achieved via the canonical Smad2/3 activation and the non-canonical pathway of *Tgf-β* activity. These results suggest that FRB may be an alternative therapeutic agent against inflammation-induced intestinal fibrosis.

## 1. Introduction

Inflammatory bowel disease (IBD) is characterized by diarrhea, bloody stools, abdominal pain, and weight loss. It is an umbrella term that can be used to refer to Crohn’s disease and ulcerative colitis (UC) [1]. Albeit more commonly found in Western countries, emerging evidence suggests that IBD is increasingly prevalent in South America, Eastern Europe, Asia, and Africa [2,3]. IBD was originally considered to manifest in genetically vulnerable individuals, and there are currently 163 loci known to contribute to the onset of IBD. Examples of these genes are *NOD2* (which is related to innate sensing of bacteria) [2,4], *IL23R* (which regulates inflammatory response to microbes) [2], *CARD9* (which integrates signals from innate immune receptors and activates different pathways of cytokines in response) [5], and *ATG16L1* (which controls autophagy) [2]. Apart from genetic factors, environmental factors such as smoking, hygiene, dietary intake, and antibiotics usage are also considered to affect the onset of IBD [4].

Various animal models have been introduced to mimic the symptoms of IBD, including chemically induced animal models using dextran sodium sulfate (DSS) [6,7,8,9] or 2,4,6-trinitrobenzene sulfonic acid [10], bacteria-induced models such as Salmonella-induced colitis [11], and genetically modified animals such as Il-10 [12] or T cell receptor α [13] knock-out mice. Among these models, DSS-induced colitis was noted to produce colitis symptoms that resemble those of UC in humans [11]. The DSS-induced model is also notable for being easy to reproduce and manipulate [14]. DSS-induced colitis in an animal model that is particularly prone to inflammation, such as C57BL/6 mice, was also noted to progress into chronic colitis instead of showing signs of mucosal healing after DSS was removed [15,16]. This animal model was suggested to be a good candidate to study the progression of acute epithelial inflammation into the subepithelial fibrosis in UC [17].

Rice bran fermented with *Aspergillus kawachii* and a mixture of lactic acid bacteria (fermented rice bran (FRB)), in particular, has been proven to be able to prevent the detrimental intestinal inflammation caused by DSS administration [7]. Islam et al. [7] initiated an FRB-supplemented diet in mice, 4 days before inducing intestinal inflammation by introducing DSS in their drinking water. FRB supplementation was found to elevate the amounts of short-chain fatty acids and tryptamine, a microbial metabolite from tryptophan in the intestine, which in turn might regulate the intestinal barrier tight junction and intestinal homeostasis. This effect was suggested to be induced partially due to the bioactive compounds of rice bran, such as polysaccharides [18], glycoproteins [19], γ-oryzanol, phytosterols, and vitamin E [20]. Fermentation of rice bran (RB) has been suggested to make its bioactive compounds more accessible and easier to metabolize [7,21,22]. A previous study has shown that fermentation enhances the amount of the total phenolic content in RB [22]. Fermentation also increases the amount of tryptophan [7], which is suggested to be able to ameliorate intestinal inflammation [8]. Therefore, it is likely that FRB supplementation can be used not only as a preventive measure but also as a therapeutic agent against an ongoing intestinal inflammation.

While a previous study already investigated the beneficial effect of FRB supplementation against the onset of DSS-induced inflammation, it was also hypothesized that this effect was partially achieved via FRB prebiotic function [7]. FRB was suggested to be able to promotes the symbiotic environment in mice intestine, thereby preventing the development and progression of DSS-induced colitis [7]. However, changes in the composition of intestinal microbiota population are commonly established in patients with UC. Furthermore, the presence of intestinal microbiota and their metabolites were shown to alter intestinal defense mechanism against mucosal inflammation [23]. Another study also reported impaired DNA integrity due to oxidation which persisted even after the intestinal inflammation has subsided [24]. Hence, the usage of FRB supplementation as a therapeutic treatment rather than a preventive measure may not work as intended. This is an important distinction to make, especially in the case of IBD, since treatments in IBD are generally aimed to prevent a flare-up of an ongoing inflammation and to hinder its progression into an irreversible state such as stricture and altered colonic motility and permeability [25].

Chronic inflammation in the intestine often results in intestinal fibrosis, as is often the case in patients with IBD. Fibrosis is characterized by excessive deposition of the extracellular matrix components, such as collagen, basement membrane proteins, and fibronectin, in response to chronic inflammation [26]. While fibrosis may be considered a part of the normal wound healing process, uncontrolled and persistent fibrosis in injured tissues can cause anatomical alterations and loss of function [27]. Intestinal fibrosis was found to cause strictures due to scar formation and tissue distortion [26,27,28,29]. IBD-associated fibrosis often leads to the loss of intestinal motility and permeability, causing loose stool or diarrhea even in the absence of inflammation [25]. Intestinal fibrosis can also develop into intestinal blockage [29], which requires treatment such as dilation and insertion of stents [30] or even surgical intervention [29]. FRB in diet has been shown to prevent the onset of intestinal inflammation [7], and, thus, it might be able to reduce the risk of intestinal fibrosis. In the current study, we observed whether dietary supplementation with FRB would be able to enhance the recovery from DSS-induced intestinal inflammation in mice. We also evaluated FRB function in preventing the onset of intestinal fibrosis due to DSS-induced chronic inflammation in mice.

## 2. Materials and Methods

### 2.1. Animals

Fifty-six female C57BL/6N mice (CLEA Japan, Inc., Tokyo, Japan) aged 11–12 weeks were used in our study. Male mice were excluded in the current study due to their high mortality rates from DSS-induced colitis [31]. The mice were housed in a pathogen-free environment at a temperature of approximately 23 ± 3 °C, relative humidity of 55 ± 10%, and a 12 h light/dark cycle. The mice had free access to their diet and drinking water throughout the experiment. The ethics approval document of this animal experiment was provided on 6 February 2018.

### 2.2. Experimental Groups, Diets, and Procedures

At the beginning of the experiment, all 56 mice were habituated with the control diet for 4 days. After 4 days, a solution of 1.5% DSS salt (MW > 40 kD, Sigma-Aldrich, St Louis, MO, USA) in tap water was used instead of the drinking water for mice. This solution was freely accessible to mice for 5 days. After 5 days, the 1.5% DSS solution was swapped with tap water. Randomization was done by assigning a randomly generated number to each individual before mice were divided into seven different groups. The mice in one group (n = 8) were euthanized with the combination of pentobarbital sodium (Kyouritsu Seiyaku Co., Tokyo, Japan) and exsanguination immediately after the DSS treatment ended. The remaining six groups were given different diets. In the current research, the supplemented diets were introduced after the DSS treatment was stopped so that the therapeutic effect of FRB could be investigated instead of its preventive one. The first group was given the control diet; the second group was fed with a diet supplemented with 10% RB; and the third group was given a diet supplemented with 10% FRB. The composition of these diets is shown in Table 1. FRB was prepared by mixing rice bran, which was fermented by *Aspergillus kawachii* at 30 °C for 44 h, and rice powder with 2:1 proportion. This mixture was added to 4 times the amount of water and saccharified by heating the resulted slurry, followed by lactic acid bacteria mixture (*Lacticaseibacillus rhamnosus*, *Levilactobacillus brevis*, and *Enterococcus faecium*) fermentation. After each fermentation, FRB was heated for 15 min at 85 °C. The fermented product was filtered, lyophilized, and stored at −30 °C until use. RB was prepared with a similar method without the inoculation of both *Aspergillus kawachii* and lactic acid bacteria. FRB and RB preparation methods were described by Islam et al. [7]. Diet treatment was conducted for 35 days, during which the body weight and disease activity index (DAI) of mice were closely monitored and scored. The scoring system to evaluate mouse DAIs is presented in Table 2. Eight mice from each group were euthanized on the 14th day after the diet treatment started. The remaining 24 mice were sacrificed at the 35th day of the diet treatment. After being euthanized, organs such as the spleen and colon were removed and measured. Samples of the spleen, colon, and blood of the mice were also obtained.

### 2.3. Quantitative Reverse Transcription Polymerase Chain Reaction

Quantitative reverse transcription polymerase chain reaction (RT-qPCR) was performed as described previously [32], with some modifications. RNA from the middle section of the colon samples was collected using the ISOGEN reagent (Nippon Gene, Co., Ltd., Tokyo, Japan) and purified further with the RNeasy mini kit (QIAGEN GmbH, Hilden, Germany) according to the manufacturer’s instructions. The isolated RNA was then used to synthesize cDNA. Synthesis of cDNA was performed by mixing the isolated RNA with 5 μM oligo-dT primer (Hokkaido System Science Co., Sapporo, Japan) and 1 mM dNTP (GE Healthcare, Tokyo, Japan) for 5 min at 65 °C. The resulting aliquot was then mixed with the RT buffer (50 mM Tris-HCl (pH 8.3), 75 mM KCl, 3 mM MgCl_2_, and 5 mM dithiothreitol) containing 50 U SuperScript III reverse transcriptase (Invitrogen, Carlsbad, CA, USA) and 20 U RNaseOUT RNase inhibitor (Invitrogen, Carlsbad, CA, USA). This mixture was then incubated at 50 °C for 60 min for reverse transcription. The resulting cDNA was then analyzed with quantitative PCR using the Bio Rad CFX Connect real-time PCR detection system (Hercules, CA, USA). Gene-specific primers (Table S1) and the TB Green Premix Ex Taq (Takara Bio, Otsu, Japan) were mixed with the cDNA aliquot to amplify the target DNA. The data obtained were then normalized to the level of eukaryotic elongation factor 1α1 (*Eef1a1*) as the internal standard.

### 2.4. Enzyme-Linked Immunosorbent Assay (ELISA)

Serum IL-6 level was evaluated with commercially available mouse ELISA kits (R&D Inc., Minneapolis, MN, USA).

### 2.5. Western Blotting

The colon samples were homogenized in phosphate-buffered saline that had been mixed with protease inhibitors (cOmplete Protease Inhibitor Cocktail; Roche Applied Science, Mannheim, Germany) and phosphatase inhibitors (PhosSTOP Phosphatase Inhibitor Cocktail, Roche Applied Science). The homogenates were then centrifuged at 12,000× *g* and 4 °C for 10 min to separate the protein lysates. Protein concentration of the lysates was measured using the Lowry protein assay. Electrophoresis of the lysates was performed using 12.5% sodium dodecyl sulfate polyacrylamide gels. The separated proteins were then transferred onto an Immobilon-P membrane (Millipore, Billerica, MA, USA). This membrane was subsequently submerged with gentle agitation in a blocking buffer, which constituted of 5% bovine serum albumin in TBS-T buffer (10 mM Tris-HCl (pH 7.4), 150 mM NaCl, and 0.1% Tween 20) for 1 h. Next, the membrane was washed with TBS-T buffer and incubated with primary antibodies against SMAD2/3 and phosphorylated SMAD2/3, overnight at 4 °C. The antibody against phosphorylated SMAD2/3 (Cell Signaling Technology, Danvers, MA, USA) was diluted in the Can Get Signal Immunoreaction Enhancer Solution (Toyobo Co., Ltd., Osaka, Japan), while the antibody against SMAD2/3 (Cell Signaling Technology) was diluted in the blocking buffer. To detect the proteins which reacted with the primary antibodies, a secondary antibody (Pierce Goat Anti-Rabbit IgG (H + L) HRP, Thermo Scientific, Rockford, IL, USA) and the Immobilon Western Detection reagent (Millipore) were used with a luminescent image analyzer (LAS-4000 mini; Fujifilm, Tokyo, Japan). A housekeeping protein, α-tubulin, was used to normalize the protein levels. Pierce ECL Western Blotting Substrate (Thermo Scientific) was used to detect the level of α-tubulin using an antibody against it (Thermo Scientific).

### 2.6. Histological Analysis

Distal colon tissues were fixed in a 10% formalin solution and then dehydrated with a succession of ethanol solutions of increasing concentrations. The dehydrated tissue samples were then embedded in paraffin, sectioned to 4 μm thickness, mounted on a glass slide, and dried. The paraffin sections were dissolved in a series of xylene solutions before staining, and the tissue sections were rehydrated with ethanol solutions of decreasing concentrations. Two different methods of staining were used: hematoxylin and eosin staining (H&E) and Heidenhain’s azan trichrome staining. H&E staining results were scored according to the degree of severity of several markers, namely, epithelial loss, crypt cell damage, and infiltration of inflammatory cells in the mucosa. The score range (for damage in the tissues) was from 0 to 4, as follows: 0, no signs of damage; 1, mild damage; 2, moderate damage; 3, severe damage; and 4, massive damage [8]. Heidenhain’s azan trichrome sections were analyzed using the software ImageJ, using the Azan-Mallory plugin. The threshold for each color was set as follows: Color 1, 110; Color 2, 200; and Color 3, 200. Four optical fields were evaluated from each slide from 7–8 specimens from each group. Histological evaluations were performed by the authors (Y.O. and T.N.) of this study.

### 2.7. Statistical Analysis

The data in this paper are expressed as a scatter plot of the data which include the median ± interquartile values. All data were tested for normality with Shapiro–Wilk test. Colon mRNA levels and serum Il-6 level data which passed this test were then further analyzed by one-way analysis of variance (ANOVA), while data which failed this test were analyzed by Kruskal–Wallis test. Tukey’s or Dunnett’s post hoc analysis was used to evaluate differences between groups. Spearman Rank Correlation test was used to determine the correlation between serum level and colon mRNA expression of Il-6. Body weight reduction and DAI were analyzed by repeated measurement ANOVA (RM ANOVA). Histological analysis scores were analyzed with one-way ANOVA, followed by Tukey–Kramer post hoc analysis. Groups were considered to be significantly different at the levels indicated on each figure. Statistical analyses were performed with the SigmaPlot software version 12.5 (San Jose, CA, USA).

## 3. Results

### 3.1. General Observation of Colitis

**Figure 1:**
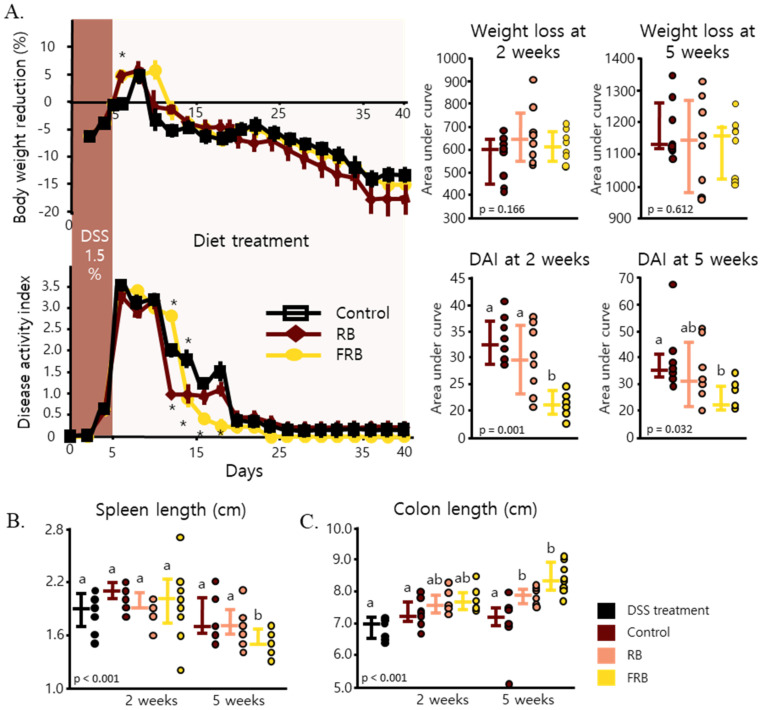
Fermented rice bran (FRB) and rice bran (RB) supplementation assist in faster recovery after dextran sodium sulfate (DSS) administration. Body weight reduction and disease activity index (DAI) were observed in the control, RB, and FRB groups, for 40 days (**A**). Spleen lengths (**B**) were measured 2 and 5 weeks after the diet treatment started. Mice colon lengths were also measured (**C**). Data regarding the body weight and DAI are presented as the mean values, with error bars representing the standard errors, and the data were analyzed via repeated measurement ANOVA and evaluated against the control group, n = 8, Dunnett’s analysis, * *p* < 0.05. Area under curve (AUC) was evaluated with Tukey’s post hoc test (*p* < 0.05, n = 8). Spleen and colon lengths were evaluated via Tukey’s analysis. The values marked with different letters (a, b) were significantly different at *p* < 0.05. AUC data and organs measurement are presented as scatter plots with bars representing the median values and the interquartile ranges.

Symptoms of DSS-induced colitis include loss of body weight, diarrhea, and presence of blood in feces [15]. Body weight loss began to be observed 2 days after the administration of DSS was stopped. In the control and RB groups, body weight loss peaked 4 days after the administration of DSS was stopped, and the maximum body weight loss was observed 5 days after the end of DSS administration, in the FRB group. After the maximum body weight loss was reached, the mice slowly regained some body weight; however, there was no significant difference observed among the three groups during the weight gain. We also observed no statistical difference in the area under the curve (AUC) value (*p* = 0.166 for 2 weeks and *p* = 0.612 for 5 weeks). These trends are illustrated in Figure 1A. The DAI scores were also observed to spike 4 days after the DSS administration was started and reached the peak one day after the DSS administration was stopped. One day post-DSS administration, the presence of blood in the feces started to decline, while diarrhea persisted until the end of the experimental period. The DAI of the RB group reduced rapidly 7 days after diet treatment was started and stayed relatively stable for another 6 days, after which it started to decrease again. The DAI of the FRB group was found to abruptly decline 9 days after the mice were given the FRB-supplemented diet, and it continued to decrease until Day 25, after which it attained a relatively steady level until the end of the experimental period. However, the control group exhibited a decline in its DAI score 7 days after DSS administration was stopped, and it continued to decline slowly for another 6 days, after which the DAI score remained relatively unchanged. AUC value for DAI after 2 weeks of FRB supplementation is lower than the other groups (*p* = 0.001), and 5 weeks of FRB supplementation also reduced this value (*p* = 0.032). It is important to note that, in comparison to a previous study [7], in which mice were administered 3% DSS for 12 consecutive days [7], the DAI scores from this study were noticeably lower, suggesting a low-grade chronic inflammation instead of a severe acute inflammation.

**Figure 2:**
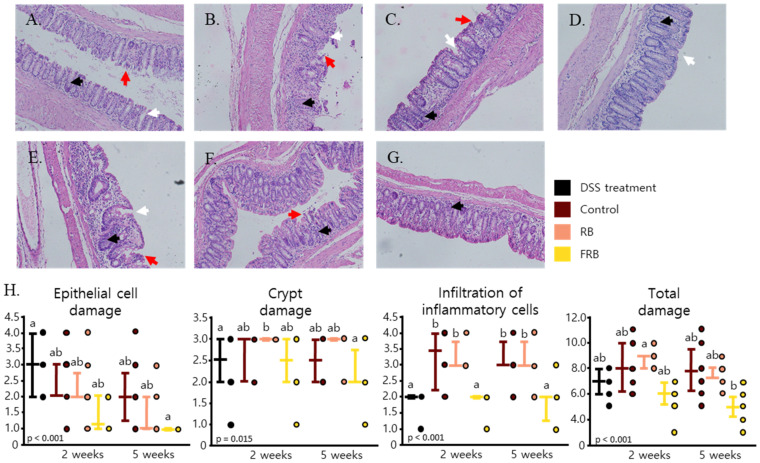
Histological sections of intestines of mice. Colon tissues from mice after the dextran sodium sulfate (DSS) administration (**A**), 2 weeks after control diet (**B**), RB-supplemented diet (**C**), and FRB-supplemented diet treatment (**D**), as well as 5 weeks after control diet (**E**), RB-supplemented diet (**F**), and FRB-supplemented diet (**G**) were stained with hematoxylin and eosin. Red arrows represent the epithelial cell damages, white arrows point to crypt damages, and black arrows indicate inflammatory cells infiltration. The sections were scored 0–4 based on the epithelial cell damage, crypt damage, and infiltration of inflammatory cells (**H**), with 0 being no signs of damage and 4 indicating massive damage. Data are presented as scatter plots with bars representing the median values and the interquartile ranges. Data were evaluated against each other, n = 7–8, Tukey–Kramer analysis. The values marked with different letters (a, b) were significantly different at *p* < 0.05. FRB, fermented rice bran; RB, rice bran.

Other symptoms of DSS-induced colitis are enlarged spleen and a decrease in the size of the cecum [15]. Figure 1B shows the change in the length of mice spleen. The difference in length of the FRB group spleens was clearly observed 5 weeks after the FRB-supplemented diet was introduced (*p* < 0.001). Colon lengths of mice from both the RB and FRB groups were found to be slightly longer 2 weeks after the diet treatment was initiated, and they significantly differed from the control group 5 weeks after the diet treatment was begun (*p* < 0.001, Figure 2C).

H&E staining was performed to evaluate the morphological changes in the intestinal lining after DSS-induced inflammation and diet treatment (Figure 2A–G). DSS-induced inflammation is often marked with changes in the epithelium, infiltration of inflammatory cells to the mucosa and sub mucosal edema, loss of crypts, and reduction of goblet cells [15,33,34,35]. These symptoms were apparent five days after DSS was administered in mice. Five days after DSS administration was started, epithelial cell damage occurred, followed by crypt damage. At this point, inflammatory cells also started to infiltrate the mucosal layer of the large intestine (Figure 2A). While signs of epithelial regeneration started to become visible 2 weeks after DSS administration was stopped; major crypt damage was also visible at this time point, and the presence of inflammatory cells in the mucosal layer was obvious (Figure 2B). Slight improvements were observed on the mucosal layer 5 weeks after DSS administration, but the signs of crypt damage and presence of inflammatory cells could still be observed (Figure 2E). FRB supplementation was found to aid in intestinal epithelial restoration (*p* < 0.001), reinstatement of intestinal crypts (*p* = 0.015), and modulation of the infiltration of inflammatory cells in the mucosal layer of the intestine (*p* < 0.001). Overall, FRB decreased the total damage (*p* < 0.001) caused by DSS-induced inflammation in the intestine (Figure 2D,G,H).

### 3.2. RB and FRB Supplementation Reduces Pro-Inflammatory Cytokine Levels and Increases Anti-Inflammatory Cytokine Levels in Mice Intestines

**Figure 3:**
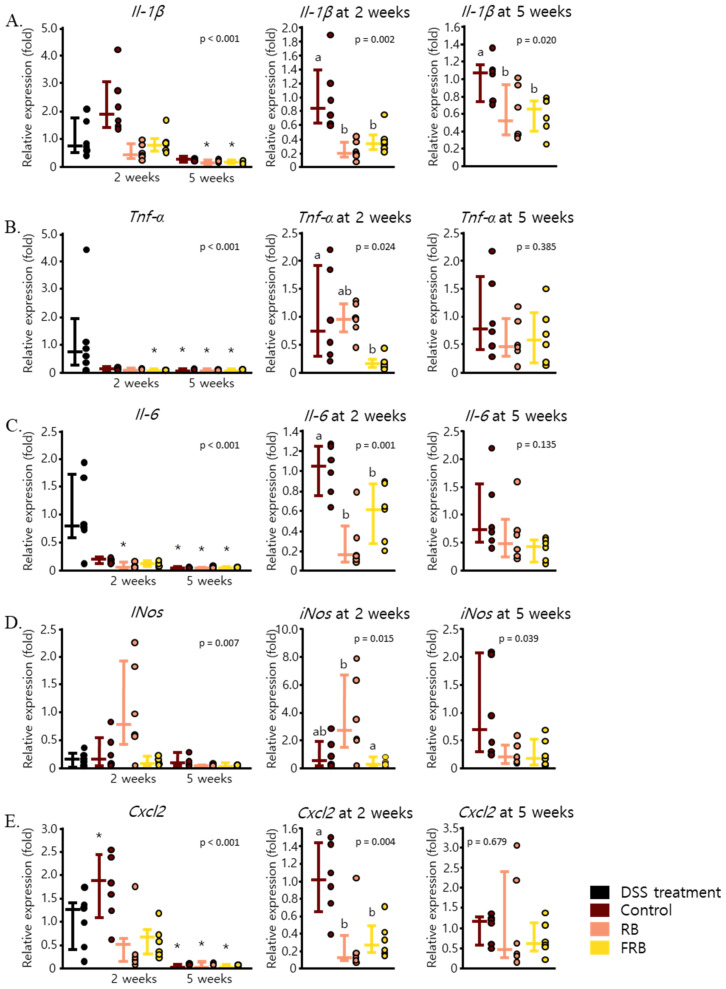
Fermented rice bran (FRB) and rice bran (RB) supplementation reduced the mRNA expression of pro-inflammatory cytokines and chemokines in mice intestines. The levels of *Il-1β* (**A**), *Tnf-α* (**B**), *Il-6* (**C**), *iNos* (**D**), and *Cxcl2* (**E**) throughout the study period and after 2 and 5 weeks of diet treatment are displayed. Data are presented as scatter plots with bars representing the median values and the interquartile ranges, n = 6. The data were evaluated against the dextran sodium sulfate (DSS) treatment group in the overall analysis (Dunnett’s analysis, * *p* < 0.05). For the values reported for 2 and 5 weeks, the data were compared against each other (Tukey’s analysis, the values marked with different letters (a, b) were significantly different at *p* < 0.05).

DSS-induced colitis in mice is often marked by the increase of pro-inflammatory cytokines. Previous studies have reported an increase in mRNA levels of *Il-1β*, *Tnf-α*, and *Il-6* [7,36] after acute colitis was induced in mice. In this study, 1.5% DSS was administered in the drinking water of mice for 5 days to cause low-grade inflammation in mice. After the DSS administration was stopped, the mice were left to recover with or without the addition of RB- or FRB-supplemented diets. The effect of DSS-induced intestinal inflammation on the production of pro-inflammatory cytokines in mice is presented in Figure 3. *Il-1β* mRNA levels escalated post-DSS administration and remained high after 5 weeks (Figure 3A). Both the RB- and FRB-supplemented diets appeared to reduce the expression of *Il-1β* at 2 weeks (*p* = 0.002) and 5 weeks (*p* = 0.002). DSS-induced mRNA level of *Tnf-α* decreased significantly in the control group 5 weeks after DSS administration was stopped (*p* < 0.001). This result is presented in Figure 3B. FRB, but not RB, supplementation further reduced *Tnf-α* expression after 2 weeks of diet treatment (*p* = 0.024). Similar to *Tnf-α*, the mRNA level of *Il-6* in the colon in control group also declined 5 weeks after the diet treatment was initiated (*p* < 0.001, Figure 3C). Nonetheless, both the RB- and FRB-supplemented diets were able to hastily ameliorate *Il-6* mRNA expression level after only 2 weeks of diet treatment (*p* = 0.001).

The upregulation of pro-inflammatory cytokines such as *Il-1β*, *Il-6,* and *Tnf-α* due to DSS-induced inflammation also contributes to the upregulation of *iNos* [34,37,38]. The mRNA level of *iNos* was found to be relatively constant throughout the 5 weeks after DSS administration was stopped (*p* = 0.007, Figure 3D). However, RB supplementation for 2 weeks increased the expression level of *iNos*, while FRB supplementation slightly decreased it (*p* = 0.015). In addition, the mRNA level of *Cxcl2*, a neutrophil-attracting chemokine [39], was enhanced after colitis was induced by DSS, and its expression level continued to be modulated 2 weeks after DSS administration was stopped (*p* < 0.001). Two weeks of diet supplementation with either RB or FRB was able to limit the expression levels of this chemokine (*p* = 0.004, Figure 3E).

**Figure 4:**
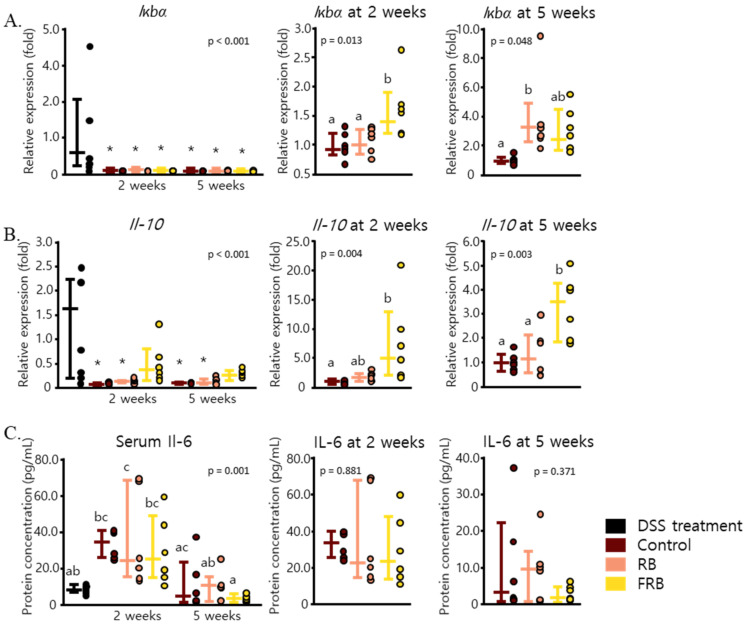
Fermented rice bran (FRB) and rice bran (RB) supplementation increased the mRNA levels of anti-inflammatory cytokines in mice intestines and the levels of pro-inflammatory proteins in serum. The levels of *Il-10* (**A**) and *Iκbα* (**B**) throughout the study period and at 2 and 5 weeks after diet treatment are presented. The amount of Il-6 protein in mice sera was also measured (**C**). Data are presented as scatter plots with bars representing the median values and the interquartile ranges, n = 6. The data were evaluated against the dextran sodium sulfate (DSS)-administered group in the overall mRNA levels analyses (Dunnett’s analysis, * *p* < 0.05). For values reported at 2 and 5 weeks, the data were compared against each other (Tukey’s analysis, the values marked with different letters (a, b) were significantly different at *p* < 0.05). For the serum *Il-6* level values, the data were compared against each other (Tukey’s analysis, the values marked with different letters (a, b, c) were significantly different at *p* < 0.05).

The effects of dietary RB and FRB supplementation in decreasing the levels of *Il-1β*, *Il-6*, and *Tnf-α* might have been due to their ability to modulate NF-ĸB, the transcription factor of these cytokines. IĸB inhibits the activity of NF-ĸB, and its kinase activation is essential to induce the response to pro-inflammatory stimuli via NF-ĸB activation [40]. FRB supplementation was able to amplify the mRNA level of *Iĸbα* after 2 weeks (*p* = 0.013), and dietary supplementation with both RB and FRB enhanced the mRNA expression of *Iĸbα* after 5 weeks (*p* = 0.048, Figure 4A). FRB supplementation also maintained the mRNA level of *Il-10*, an anti-inflammatory cytokine (*p* < 0.001), as shown in Figure 4B. While Il-6 is thought to be involved as a pro-inflammatory cytokine in the pathogenesis of IBD, this cytokine has also been reported to play a role in intestinal mucosal healing [41]. The protein level of *Il-6* in mice sera was found to be elevated 2 weeks after DSS administration was stopped (*p* = 0.001, Figure 4C). The level of Il-6 in mice sera was found to be positively correlated with its mRNA level (*p* = 0.00244). Consistent with its colon mRNA expression, FRB supplementation for 5 weeks was able to reverse the upsurge in the levels of *Il-6*.

### 3.3. Effect of RB and FRB Supplementation on Intestinal Barrier Function

**Figure 5:**
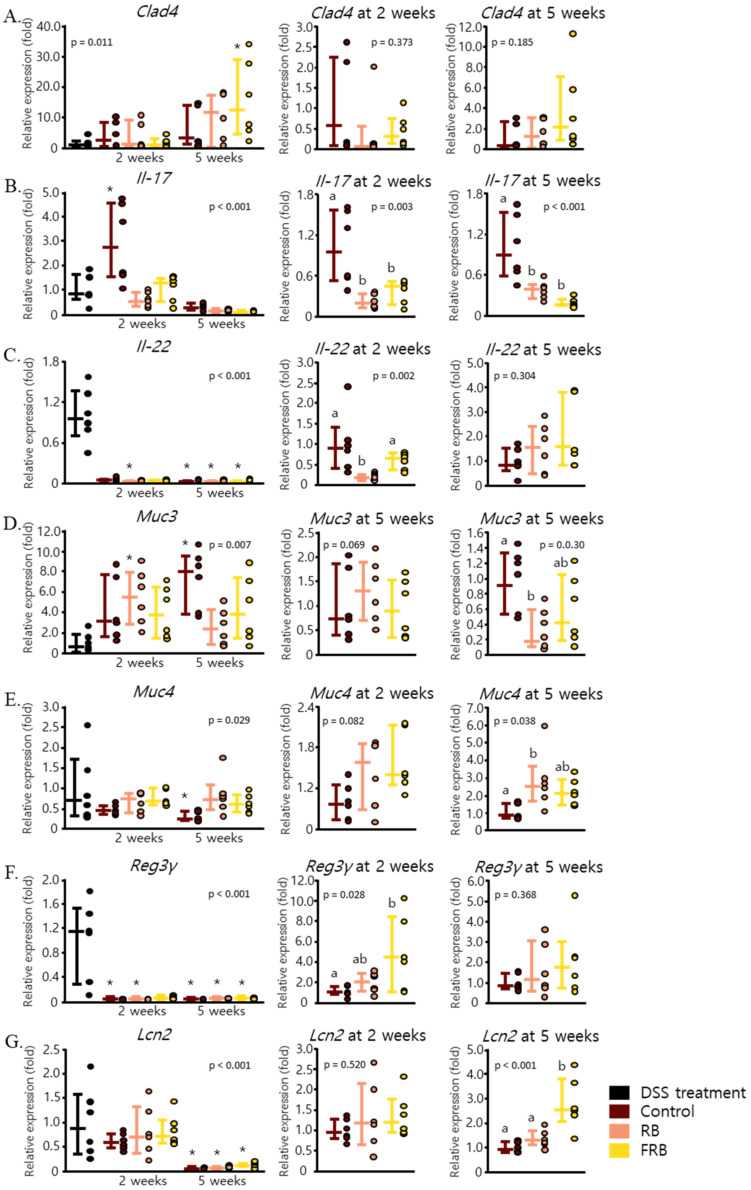
Fermented rice bran (FRB) and rice bran (RB) supplementation regulated intestinal barrier function. RB and FRB attenuated the expression levels of intestinal barrier markers *Clad4* (**A**), *Il-17* (**B**), and *Il-22* (**C**). The mRNA levels of antibacterial peptides *Muc3* (**D**), *Muc4* (**E**), *Reg3γ* (**F**), and *Lcn2* (**G**) throughout the study period and after 2 and 5 weeks of diet treatment are presented. Data are presented as scatter plots with bars representing the median values and the interquartile ranges, n = 6. The data were evaluated against the dextran sodium sulfate (DSS)-administered group in the overall analysis (Dunnett’s analysis, * *p* < 0.05). For values reported at 2 and 5 weeks, the data were compared against each other (Tukey’s analysis, the values marked with different letters (a, b) were significantly different at *p* < 0.05).

Figure 5A displays the effect of dietary FRB supplementation in restoring the intestinal barrier by enhancing the mRNA level of *Clad4*, a component of the intestinal tight junction [42], after 5 weeks of diet treatment (*p* = 0.011). Furthermore, FRB was able to mitigate the mRNA level of *Il-17* (*p* = 0.003 at 2 weeks and *p* < 0.001 at 5 weeks, Figure 5B), a cytokine which is commonly produced by T helper (Th17) cells and plays the role of an important mediator in the pathogenesis of colitis [43,44]. *Il-22* is a cytokine that belongs to the Il-10 family and is produced by various immune-related cells, including innate lymphoid cells, at sites of inflammation. The presence of *Il-22* promotes tissue restoration in the intestine and induces the secretion of antimicrobial peptides [45,46,47]. FRB treatment maintained the level of *Il-22* mRNA 2 weeks after DSS administration was ceased (*p* = 0.002, Figure 5C). FRB supplementation also maintained the mRNA levels of antimicrobial peptides *Reg3γ* (*p* < 0.001) and *Lcn2* (*p* < 0.001 at 5 weeks), but the expression of *Muc3* (*p* = 0.003) and *Muc4* (*p* = 0.038) was only slightly affected (Figure 5D–G).

### 3.4. RB and FRB Supplementation Regulates Extracellular Matrix Deposition in the Intestine

**Figure 6:**
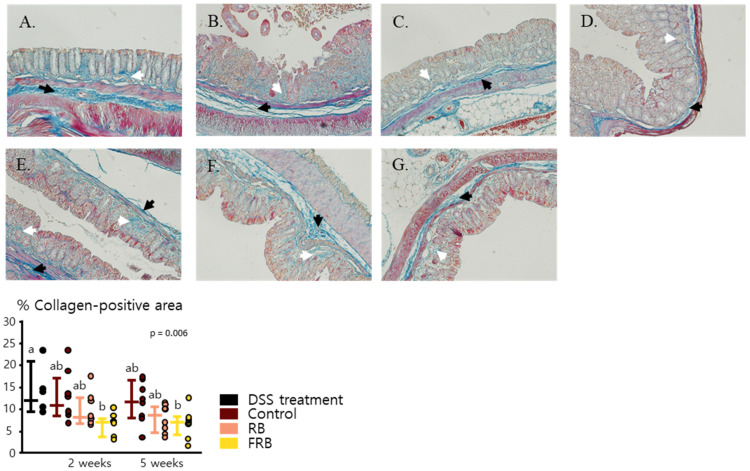
Fermented rice bran (FRB) supplementation reduced collagen deposition in mice colon. The Heidenhain’s azan trichrome staining of large intestine sections from each group are presented. After DSS treatment was stopped (**A**), 2 weeks after control diet (**B**), RB-supplemented diet (**C**), and FRB-supplemented diet (**D**) administration, as well as 5 weeks after control diet (**E**), RB-supplemented diet (**F**), and FRB-supplemented diet (**G**) treatment. The collagen-positive area (black arrows indicate the collagen deposition in submucosal, and white arrows represent collagen deposition in lamina propria) was analyzed against the total stained area. Data are presented as scatter plots with bars representing the median values and the interquartile ranges, n = 7–8. The data were evaluated against the dextran sodium sulfate (DSS)-administered group in the overall analysis (Tukey–Kramer analysis, the values marked with different letters (a, b) were significantly different at *p* < 0.05).

**Figure 7:**
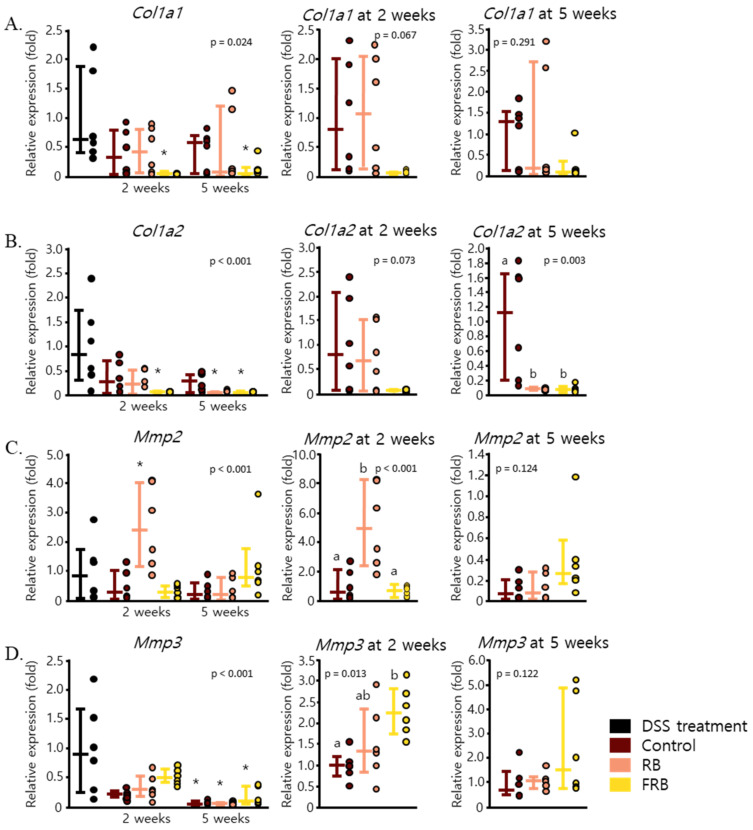
Fermented rice bran (FRB) supplementation reduced markers of intestinal fibrosis. The levels of *Col1a1* (**A**), *Col1a2* (**B**), *Mmp2* (**C**), and *Mmp3* (**D**) throughout the study period and at 2 and 5 weeks after diet treatment are presented. Data are presented as scatter plots with bars representing the median values and the interquartile ranges, n = 6. The data were evaluated against the dextran sodium sulfate (DSS)-administered group, in the overall analysis (Dunnett’s analysis, * *p* < 0.05). For values reported at 2 and 5 weeks, the data were compared against each other (Tukey’s analysis, the values marked with different letters (a, b) were significantly different at *p* < 0.05).

Chronic colitis often progresses into fibrosis, which is characterized by excessive collagen deposition in all layers of the intestinal wall [15]. In this study, DSS-induced colitis was observed to enhance collagen deposition in the large intestine, as presented in Figure 6A–G, which displays the large intestine section that was stained via Heidenhain’s azan trichrome staining. The FRB group was observed to show less collagen compared to the control and RB groups (*p* = 0.006, Figure 6). FRB supplementation also showed reduced mRNA levels of *Col1a1* (*p* = 0.024) and *Col1a2* (*p* < 0.001), as seen in Figure 7A,B. Collagen 1 is known as one of the markers of collagen deposition in the intestinal lining [48,49]. The presence of the extracellular matrix in tissue is regulated by matrix metalloproteinases, which suggests that the regulation of these enzymes plays a role in the onset of fibrosis [50]. Two weeks of dietary RB supplementation also increased the mRNA level of *Mmp2* (*p* < 0.001, Figure 7C), while RB and FRB supplementation elevated the mRNA level of *Mmp3* (*p* = 0.013, Figure 7D). Mmp-2 is known as gelatinase and is able to degrade denatured collagen. Its presence is essential for intestinal protection against DSS-induced colitis [51]. Mmp-3 is stromelysin; able to degrade proteoglycans, collagens, laminin, fibronectin; and can activate collagenase [52]. The presence of Mmp-3 in gastrointestinal ulcer possibly affects tissue repair at multiple stages [52].

**Figure 8:**
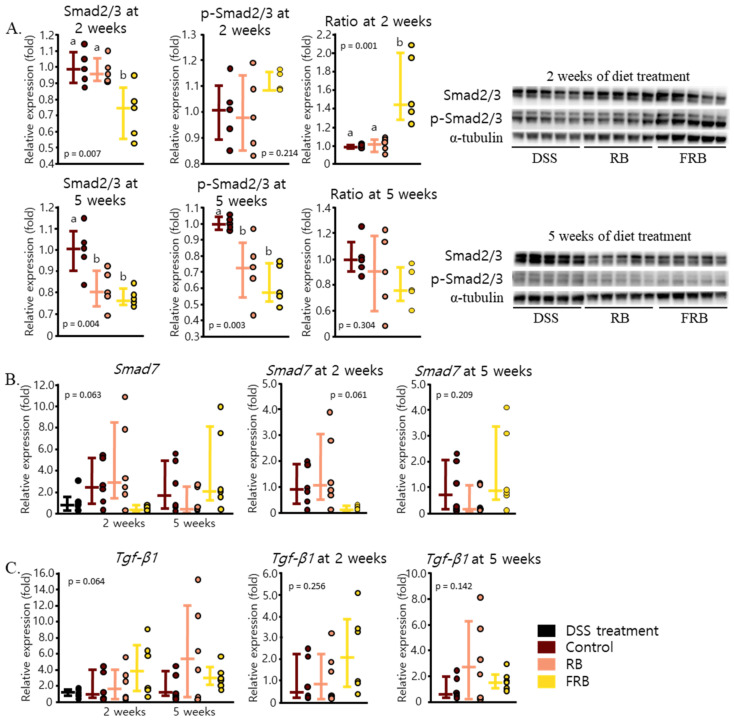
Fermented rice bran (FRB) supplementation reduces the risk of intestinal fibrosis. The levels of Smad2/3 proteins and their phosphorylated forms were measured at 2 and 5 weeks of diet treatment (**A**). The mRNA level of *Smad7*, an inhibitor of Smad2/3 (**B**) and *Tgf-β1* (**C**), were also measured (**B**). Data are presented as scatter plots with bars representing the median values and the interquartile ranges (n = 5 for protein analysis and n = 6 for mRNA analysis). Protein levels were evaluated with Tukey’s analysis, and the values marked with different letters (a, b) were significantly different at *p* < 0.05. The mRNA levels were evaluated against the dextran sodium sulfate (DSS)-administered group, in the overall analysis, and against the control group for the 2- and 5-week reports (Dunnett’s analysis).

During intestinal fibrosis, collagen production is controlled by the activity of Smad2/3, a downstream signaling target of *Tgf-β* [53]. As illustrated in Figure 8A, 2 weeks of FRB supplementation decreased the protein level of Smad2/3 (*p* = 0.007). Five weeks of FRB supplementation also decreased the level of Smad2/3 (*p* = 0.004). A slight decrease in the mRNA level of *Smad7*, an inhibitor of Smad2/3, after 2 weeks of FRB supplementation was found, as presented in Figure 8B (*p* = 0.061). The level of *Smad7* 5 weeks after FRB supplementation seemingly increased (*p* = 0.209, Figure 8B). We also found no statistical difference in the levels of *Tgf-β1* mRNA expression between all groups (*p* = 0.064, Figure 8C). These results suggest that RB and FRB supplementation might be able to prevent intestinal fibrosis due to DSS-induced inflammation via the regulation of Smad 2/3 protein level.

## 4. Discussion

The progression of DSS-induced inflammation and its development into intestinal fibrosis were observed in the current study. The presence of blood in the stool and diarrhea were observed on the fourth day after DSS administration was started (Figure 1A). The mRNA levels of pro-inflammatory cytokines such as *Il-1β, Tnf-α*, and *Il-6* (Figure 3A–C) were found to increase after the administration of DSS was ceased, and their levels significantly decreased 5 weeks after DSS administration ended. Signs of epithelial loss were visible soon after DSS administration ended; however, its detrimental effect was at its peak 2 weeks after DSS administration was stopped (Figure 2B). Five weeks after stopping the DSS administration, diarrhea was still found in the control group, and signs of crypt dysplasia were still visible, albeit intestinal epithelial regeneration had begun (Figure 1A and Figure 2E). This progression of DSS-induced colitis has been observed in other studies [54,55,56]. DSS has been shown to form nanometer-sized vesicles with medium-chain-length fatty acids in the colon upon ingestion. These complexes fuse with the intestine cell membrane and deliver DSS into the cells, thereby disrupting the activity of the cells [54]. DSS-induced inflammation starts to manifest several days after its ingestion and is marked by the spike of *Tnf-α* in the colon [55]. Tnf-α, followed by mucin depletion, leads to the erosion of intestinal epithelia, infiltration of inflammatory cells, and the upregulation of other pro-inflammatory cytokines such as *Il-1β*, *Il-6*, and *Il-17* at a later stage, along with crypt dysplasia [15,56]. Blood was visible in the stool several days after DSS-induced inflammation occurred, although it disappeared soon after DSS administration was stopped [15]. On the other hand, diarrhea occurs at about the same time as the occurrence of fecal blood, and it persists weeks after the removal of DSS [15,48]. After stopping the administration of DSS, the intestinal epithelial layer started to recover, the amount of inflammatory cell infiltration decreased, and the intestinal crypts were regenerated. The levels of *Il-1β*, *Il-6*, and *Il-17* have been observed to remain high even after the removal of DSS [15,48,56].

The mRNA level of *Il-10* (Figure 4B) and *Il-22* (Figure 5C) were also found to be at their peak after the DSS treatment was discontinued. The increase of *Il-10* and *Tgf-β*, as anti-inflammatory cytokines, in the early days of DSS-induced inflammation has been described in a previous study, and their expression indeed decreased with the progression of colitis [55]. *Tgf-β* is a pleiotropic cytokine, and its expression was reported to be increased in IBD tissues. It has been suggested that this cytokine is necessary to suppress inflammation in UC and promotes intestinal recovery [57]. In the recovering intestine, *Tgf-β1* promotes the differentiation of fibroblasts into myofibroblasts by enhancing α-smooth muscle actin, modulating myofibroblast migration, and mediating myofibroblast extracellular matrix (ECM) production [49,58]. We found no statistical differences in the mRNA levels of *Tgf-β1* with diet treatments among all the groups (Figure 8C); however, the combined effect of *Tgf-β* and other cytokines and chemokines might have been a crucial factor in the deposition of ECM in the intestinal lining, which will affect the onset of fibrosis after DSS-induced inflammation.

Excessive deposition of ECM is considered to be one of the reasons for colon thickening and shortening after the onset of DSS-induced inflammation [15,48]. Following the administration of DSS, ECM deposition was apparent in the intestine (Figure 6A). This symptom of disease progression has also been examined in other studies, in which ECM deposition was reported to be apparent in colon lamina propria, especially in the sites of severe crypt damage and inflammation after a single DSS cycle [16]. Collagen deposition was also noted in submucosa and even in the focal region of the serosa layer during the recovery stage after a single administration of DSS treatment [15,16]. Since excessive collagen deposition may lead to the development of intestinal fibrosis, FRB seemingly reduced the risk of fibrosis due to DSS-induced colitis, by reducing the mRNA expression of *Col1a1* and *Col1a2* and increasing the level of *Mmp3* (Figure 7A,B,D).

There are several ways through which *Tgf-β* exerts its fibrogenic function. The canonical pathway of *Tgf-β* signaling in fibrosis utilizes the activity of Smad2/3, and the non-canonical pathway involves the JAK1–STAT3 axis [59,60]. Dietary FRB supplementation decreased the level of Smad2/3 after 2 weeks. A longer period of supplementation decreased the levels of Smad2/3 in FRB group, yet increased its levels in RB group (Figure 8A). FRB supplementation decreased the mRNA level of *Smad7* after 2 weeks and increased it back after 5 weeks of diet treatment, albeit this was not statistically significant (Figure 8B). From these results, we can speculate that FRB supplementation is able to attenuate DSS-induced inflammation and the onset of fibrosis via the canonical pathway of *Tgf-β* and Smad2/3 activation.

FRB might also affect the non-canonical pathway of *Tgf-β*, as FRB supplementation sustained the mRNA levels of *Il-22* (Figure 5C) and *Il-10* (Figure 4B) and lowered those of *Tnf-α* (Figure 3B). *Il-22* promotes intestinal epithelial regeneration and wound healing, and therefore it also affects the development of intestinal fibrosis [61,62]. Furthermore, *Il-22* upregulates many antimicrobial proteins including Reg3γ and mucin via the expression of Stat3 [8,63]. *Il-22* is known to have a synergistic effect with *Tgf-β*, in increasing the proliferation of myofibroblasts and regulating the production of collagen [61]. Additionally, Il-10 promotes the production of intestinal mucus by suppressing endoplasmic reticulum stress via attenuation of Stat1 and Stat3 [64]. The STAT3-IL-10-IL-6 pathway has also been shown to regulate macrophage phenotype from pro-inflammatory to restorative [65]. Similarly, *Tnf-α* has been noted to promote collagen production via Tnfr2 and Stat3. The STAT3-IL-10-IL-6 pathway leads to the regulation of Timp-1 and Mmp2 activities, and therefore it affects collagen degradation. Additionally, *Tnf-α* also interacts with Igf-1 to enhance myofibroblasts proliferation, which results in collagen accumulation [66]. Therefore, it is understandable how FRB supplementation, which resulted in decreasing the level of *Tnf-α* and maintaining the levels of *Il-10* and *Il-22*, might have prevented collagen deposition and lowered the risk of fibrosis.

FRB supplementation also lowered the mRNA level of *Il-17* in the colon (Figure 5B). The elevated level of *Il-17* and increased levels of other profibrogenic cytokines such as *Il-1β*, *Tgf-β*, and *Tnf-α* were suggested to be involved in the pathogenesis of intestinal fibrosis [50]. While *Tgf-β1* is shown to regulate Th17 development and differentiation, *Il-17* is also hypothesized to independently regulate the balance of matrix metalloproteinases and the tissue inhibitor of metalloproteinase (Mmp/Timp). This hypothesis was supported by a report which stated that anti-*Il-17* antibody treatment not only lowers the expression of *Il-1β*, *Tgf-β*, and *Tnf-α* but also partially modulates the Mmp/Timp balance, indicating that other mechanisms must have been involved in the pathogenesis of intestinal fibrosis [50].

Probiotic-derived soluble compounds which are either secreted by bacteria or released after bacterial lysis, often referred to as postbiotics, have been suggested as a novel strategy in maintaining the intestinal homeostasis. Previously, a protein secreted by *Lacticaseibacillus rhamnosus* GG was reported to be able to protect mice from 3% DSS-induced colitis [67]. GABA, protein p40, lactocepin 31, and other uncharacterized soluble molecules produced from *Levilactobacillus brevis* BGZLS10-17 have also been suggested to modulate the immune response of Concanavalin A-stimulated mesenteric lymph node cells [68]. Islam et al. [7] stated that the level of tryptamine, a microbial metabolite of tryptophan, increased to about 10 times in fermented rice bran compared to non-fermented rice bran. Tryptamine, indole, and indole-acetic acid are secondary metabolites of gut microbiota, which can also function as ligands for the aryl hydrocarbon receptor (Ahr) [69]. Ahr activity has been shown to alleviate colitis by regulating the levels of *Il-22* and *Il-17*, and therefore Ahr has been suggested to maintain intestinal homeostasis by regulating the activities of intraepithelial lymphocyte, Treg, and Th17 cells [8]. As dietary FRB supplementation was able to alter the mRNA expression of both *Il-22* and *Il-17* (Figure 5B,C), it is possible that the tryptophan metabolites play a synergistic role with other bioactive compounds in FRB in ameliorating DSS-induced colitis and, subsequently, contribute to the prevention of the onset of fibrosis. Alauddin et al. [22] also remarked that fermentation process altered the macronutrients composition of RB and FRB. Fermentation increased the amount of protein (RB, 14.4 g/100 g; FRB, 15.4 g/100 g), lipid (RB, 6.7 g/100 g; FRB, 10.0 g/100 g), and dietary fiber (RB, 4.4 g/100 g; FRB, 22.0 g/100 g). On the contrary, fermentation decreased the amount of carbohydrate (RB, 49.3 g/100 g, FRB, 13.1 g/100 g). These macronutrients alterations marked the activity of microorganisms that were used in the fermentation process. However, this estimation was performed by neglecting the biomass of microorganisms which is contained in FRB after the fermentation process. As the current study did not include a group in which mice were given supplementation of the mixture of *Aspergillus kawachii*, *Lacticaseibacillus rhamnosus*, *Levilactobacillus brevis*, and *Enterococcus faecium,* without the addition of rice bran, our study could not exclude the possibility that the presence of inactive microorganisms in FRB might have played a role in FRB function in assisting the intestinal recovery or preventing the onset of fibrosis post-DSS-induced colitis. This limitation of the current study should be addressed in future studies related to FRB.

## 5. Conclusions

Dietary FRB supplementation was able to aid in intestinal restoration after DSS-induced inflammation in mice. FRB supplementation was able to reduce the DAI score and production of intestinal pro-inflammatory cytokines. FRB supplementation also increased intestinal mRNA levels of anti-inflammatory cytokine *Il-10*, tight junction component *Clad4*, and anti-microbial proteins. It was also able to prevent the development of fibrosis in mice intestines post-inflammation due to its ability to ameliorate intestinal inflammatory responses and modulate both the canonical and non-canonical pathways of *Tgf-β* profibrogenic activity. The findings of this research suggest that the usage of FRB supplementation is not limited to preventing inflammation, but it also helps the intestinal restoration in ongoing colitis. While FRB has been suggested to function as a prebiotic in the intestine, it can still ameliorate colitis even after intestinal dysbiosis potentially occurred after inflammation happened. However, the effect of FRB supplementation on the population and composition of intestinal microbiota in normal and inflamed intestines still needs to be investigated.

## Figures and Tables

**Table 1:** Diet composition (based on the AIN-93M diet composition).

Composition	Control Diet (g)	10% RB Supplemented Diet (g)	10% FRB Supplemented Diet (g)
Tert-butylhydroquinone	0.008	0.0072	0.0072
L-Cystine	1.8	1.62	1.62
Choline bitartrate	2.5	2.25	2.25
Vitamin mixture	10	9	9
Mineral mixture	35	31.5	31.5
Soybean oil	40	36	36
Cellulose	50	45	45
Sucrose	100	90	90
Casein	140	126	126
Cornstarch	620.7	558.6228	558.6228
Rice bran	-	100	-
Fermented rice bran	-	-	100
Total	1000	1000	1000
Calories per 100 g (kcal)	385	378	367

RB, non-fermented rice bran; FRB, fermented rice bran.

**Table 2:** Disease activity index score description.

Score	Diarrheal Score	Bloody Stool Score
0	Normal stool	Normal colored stool
1	Mildly soft stool	Brown stool
2	Very soft stool	Reddish stool
3	Watery stool	Bloody stool

## Data Availability

Data is contained within the article.

## Supplementary Materials

The following are available online at https://www.mdpi.com/article/10.3390/nu13061869/s1, Table S1: List of nucleotide sequences using RT-qPCR.
