# Supplementary material for: Fermented Rice Bran Supplementation Prevents the Development of Intestinal Fibrosis Due to DSS-Induced Inflammation in Mice
Source: Nutrients. 2021 May 30;13(6):1869. doi: 10.3390/nu13061869 (PMC8229226; doi:10.3390/nu13061869)
Supplement: Supplementary file 1 [file nutrients-13-01869-s001.zip › nutrients-1212946-supplementary.pdf]

Supplementary Table S1. List of nucleotide sequences using RT-qPCR

Gene name	Forward primer	Reverse primer
Eukaryotic translation elongation factor 1α1 (*Eef1a1*)	GATGGCCCCAAATTCTTGAAG	GGACCATGTCAACAATGGCAG
Interleukin-1β (*Il-1β*)	CTGTGTCTTTCCCGTGGACC	CAGCTCATATGGGTCCGACA
Tumor necrosis factor α (*Tnf-α*)	GACGTGGAACTGGCAGAAGAG	TCTGGAAGCCCCCCATCT
Interleukin-6 (*Il-6*)	AGAGGAGACTTCACAGAGGATACCA	AATCAGAATTGCCATTGCACAAC
Inducible nitric oxide synthase (*iNos*)	CAGGTGCACACAGGCTACT	GAGCACGCTGAGTACCTCATT
Chemokine (C-X-C motif) ligand 2 (*Cxcl2*)	CCAACCACCAGGCTACAGG	GCGTCACACTCAAGCTCTG
Nuclear factor of kappa light polypeptide gene enhancer in B-cells inhibitor, alpha (*Iκbα*)	CTTGGGTGCTGATGTCAATG	ACCAGGTCAGGATTTTGCAG
Interleukin 10 (*Il-10*)	TGAATTCCCTGGGTGAGAAGCTGA	TGGCCTTGTAGACACCTTGGTCTT
Claudin 4 (*Cldn-4*)	CCTCTGGATGAACTGCGTGGTG	GTCGCGGATGACGTTGTGAG
Mouse interleukin 17 (*Il-17*)	CTCCAGAAGGCCCTCAGACTAC	GCTTTCCCTCCGCATTG ACACAG
Mouse interleukin 22 (*Il-22*)	GGAGACAGTGAAAAAGCTTG	AGCTTCTTCTCGCTCAGACG
Mucin 3 (*Muc3*)	CGTGGTCAACTGCGAGAATGG	CGGCTCTATCTCTACGCTCTCC
Mucin 4 (*Muc4*)	CAGCAGCCAGTGGGGACAG	CTCAGACACAGCCAGGGAACTC
Regenerating islet-derived protein 3 gamma (*Reg3γ*)	TTCCTGTCCTCCATGATCAAAA	CATCCACCTCTGTTGGGTTCA
Lipocalin 2 (*Lcn2*)	AATGTCACCTCCATCCTGGT	CCCTGGAGCTTGGAACAAAT
Transforming growth factor beta 1 (*Tgf-β1*)	TAAAGAGGTCACCCGCGTGCTAAT	ACTGCTTCCCGAATGTCTGACGTA
Mothers against decapentaplegic homolog 7 (*Smad7*)	GTGTTGCTGTGAATCTTACGGG	CATTGGGTATCTGGAGTAAGGAG
Collagen, type I, alpha 1 (*Col1a1*)	ATCAGCTGGAGTTTCCGTGC	GGACCCATTGGACCTGAACC
Collagen, type I, alpha 2 (*Col1a2*)	ATCCGGTAACAAGGGTGAGC	GAACCAGGGCTGCCTCTAAG
Matrix metalloproteinase-2 (*Mmp2*)	CCTGTTCAACGGTCGGGAAT	GGTAAACAAGGCTTCATGGGG
Matrix metalloproteinase-3 (*Mmp3*)	CACTCCCTGGGACTCTACCA	GGGAGTTCCATAGAGGGACTG
